# A Bayesian model for estimating with-in host *P. falciparum* haplotype frequencies

**DOI:** 10.1186/1475-2875-11-S1-P36

**Published:** 2012-10-15

**Authors:** Aimee R Taylor, Jennifer A Flegg, Philippe J Guerin, Cally Roper, Chris Holmes

**Affiliations:** 1 Worldwide Antimalarial Resistance Network (WWARN), Asia Regional Centre, Bangkok, Thailand; 2 Centre for Tropical Medicine, Nuffield Department of Clinical Medicine, University of Oxford, Oxford, UK; 3 London School of Hygiene and Tropical Medicine, London, UK; 4 Department of Statistics, University of Oxford, Oxford, UK

## 

The necessity for effective surveillance of antimalarial resistance is all the more reinforced by the recent emergence of resistance to artemisinin derivatives. However, the accurate detection of resistant haplotypes from patient samples that are infected with multiple parasite clones is nontrivial. When the multiplicity of infection exceeds one, the allele sequences of the constituent clones at genotyped loci are convoluted. Nevertheless, statistical methods can be used to reconstruct the allele sequences, infer distinct haplotypes and ascertain their frequencies from prevalence data. We have developed a Bayesian model for estimating haplotype frequencies. The model estimates haplotype frequencies based on prevalence data collected for one or more molecular markers known to been associated with antimalarial resistance. Prior knowledge of the MOI is not required. The model uses a Metropolis-Hasting Monte Carlo Markov chain algorithm to explore the different possible haplotype compositions that are compatible with the sample observed, and calculates the likelihood of the data given the current estimate of the haplotype frequencies. For each haplotype the model returns a distribution of frequency estimates from which the mean and its credible interval are derived. For each sample the model returns a distribution, over the possible haplotype compositions with which it is compatible. The model is validated using simulated data sets for which the true haplotype estimates are known. We present results of the application of our model to estimate haplotype frequencies for a set of historic data from Africa in which the prevalence of mutations associated with sulphadoxine-pyrimethamine resistance were obtained.

